# Bridging Clinical Expertise and Digital Innovation: Toward a Modern Phenomenology of Parkinsonism

**DOI:** 10.1002/mdc3.70195

**Published:** 2025-07-08

**Authors:** Genko Oyama

**Affiliations:** ^1^ Department of Neurology Saitama Medical University Saitama Japan

**Keywords:** parkinsonism, digital device, artificial intelligence

The clinical diagnosis and evaluation of Parkinsonism has long relied on phenomenological assessments and expert observation. However, we now stand at a critical inflection point in neurology. The convergence of traditional clinical skills with digital and artificial intelligence (AI)‐driven tools may complement our clinical skills. This review highlights how the integration of digital biomarkers and AI‐based analysis can refine and augment our evaluation and management of Parkinsonism ushering in a new era of “modern phenomenology.”

## Current Practice in Evaluating Parkinsonism

Cardinal features of parkinsonism include tremor, rigidity, bradykinesia/akinesia, and postural instability. Parkinsonism is clinically defined as bradykinesia accompanied by either resting tremor, rigidity, or both, according to the Movement Disorder Society (MDS) criteria.^1^ The current standard for the evaluation of Parkinsonism is the MDS sponsored Unified Parkinson's Disease Rating Scale (MDS‐UPDRS).^2^ The MDS‐UPDRS consists of four parts, and Part III includes physician evaluation of motor symptoms, and it remains the gold standard for evaluating motor symptoms in Parkinson's disease (PD). Although the scale is validated and has been shown to have low inter‐rater variability, it still requires training to use and allows variability based on a physician's subjective evaluation.

## The Rise of Digital Tools

Advances in technology have led to the increasing use of various digital tools to complement clinical assessments for Parkinsonism. Each cardinal symptom traditionally evaluated through clinician observation can now be supported by objective digital measurements.

Speech can be evaluated not only with classical voice recorders but also through speech recognition software using Automatic Speech Recognition (ASR) and Natural Language Processing (NLP). Digital speech biomarkers such as voice quality, pitch, loudness, and articulation can monitor disease progression, detect the effects of treatment, and provide feedback for speech therapy.^3^ More recently, Large Language Models (LLMs) such as ChatGPT have demonstrated an enhanced ability to analyze not just acoustic features, but also linguistic content, offering novel insights into both the motor and cognitive aspects of PD.^4^, ^5^

Facial expression, a revealing marker of Parkinsonism, can now be analyzed using AI‐powered facial recognition applications.^6^, ^7^ These tools provide an objective, reproducible method to detect hypomimia, which is often difficult to measure objectively in standard clinical exams. Combining speech and facial features may further enhance the detection of neurodegenerative disease.^8^ A tablet application that evaluates both facial expression and speech during conversations with a chatbot shows high accuracy to diagnose Alzheimer's disease and PD from healthy controls.

Akinesia/bradykinesia and tremor can be measured using motion capture and video analysis systems, which can be used to measure speed and range of movement of arm and legs. These systems enable quantification of repetitive motor tasks, such as finger taps, pronation‐supination movements, hand open‐close, and tremors. Video rating is a relatively traditional method for evaluating movement disorders. Recent advances in AI technology have enhanced automated motor evaluation. A study demonstrated that AI‐based application detecting tremor and bradykinesia had high agreement rate with a good level of consistency between the gold standard expert rating and the machine learning model.^9^

Gait and posture disturbances are hallmark features of parkinsonism, traditionally assessed through clinical observation. Advancements in digital technology have introduced objective, quantifiable methods to evaluate gait and posture, enhancing both diagnostic precision and patient monitoring. For instance, 3D motion capture systems using markers and laboratory settings offer detailed gait analysis.^10^ In addition, Red‐green‐blue‐depth (RGB‐D) cameras also provide markerless 3D motion analysis for gait in PD, which might be easier to use in clinical settings.^11^

Wearable sensors, such as accelerometers and gyroscopes, have become instrumental in capturing detailed gait parameters. These devices can measure stride length, gait speed, cadence, and variability. For instance, studies have demonstrated that wearable sensor‐based gait analysis can detect subtle gait alterations in early PD subtypes, offering potential biomarkers for early diagnosis.^12^

Smartphones and smartwatches can collect continuous data, allowing for the assessment of motor fluctuations and the detection of freezing of gait episodes. Research indicates that smartwatches can help detect PD symptoms earlier by monitoring subtle shifts in movement speeds.^13^ Indeed, the Apple Watch provides an Application Programming Interface (API) that can distinguish between tremor.^14^ However, interpreting an off‐period using a wearable device remains a challenge due to ambiguity in differentiating rest from hypokinetic states. A possible solution is to offer active motor tasks during the day. A previous study showed significant sensitivity to levodopa induced change, supporting their value in home‐based monitoring.^15^ Another possible solution is to use non‐motor parameters such as pulse rate, in combination with motor parameters to identify the off‐periods.^16^

Beyond wearables, recent advancements in wireless monitoring may offer a more stress‐free way to monitor gait in PD. Wireless monitoring using radio frequency, which tracks the patient's movement throughout the day within the house, has shown that gait speed extracted from the movement data may serve as a biomarker of wearing off.^17^ Thus, wireless monitoring offers continuous assessment of PD patients without the need to wear any device.

Rigidity remains a difficult symptom to quantify, but digital tools such as electromyography (EMG), torque sensors, and accelerometers have shown potential in capturing muscle resistance objectively across joint movements.^18^

## The Future: AI And Diagnostic Redefinition

With AI‐based analysis now achieving diagnostic accuracies approaching 90%,^19^ we must ask: Can machines surpass human clinical judgment? While promising, current AI models are typically benchmarked against clinician‐derived labels as a gold standard, which limits their independence and accuracy. But how accurate are we, as physicians, in our diagnoses? For instance, a study using the Netherlands Brain Bank revealed that even expert clinical diagnosis using MDS criteria has limitations, with postmortem analyses showing a 15% misdiagnosis rate.^20^

**Figure 1:**
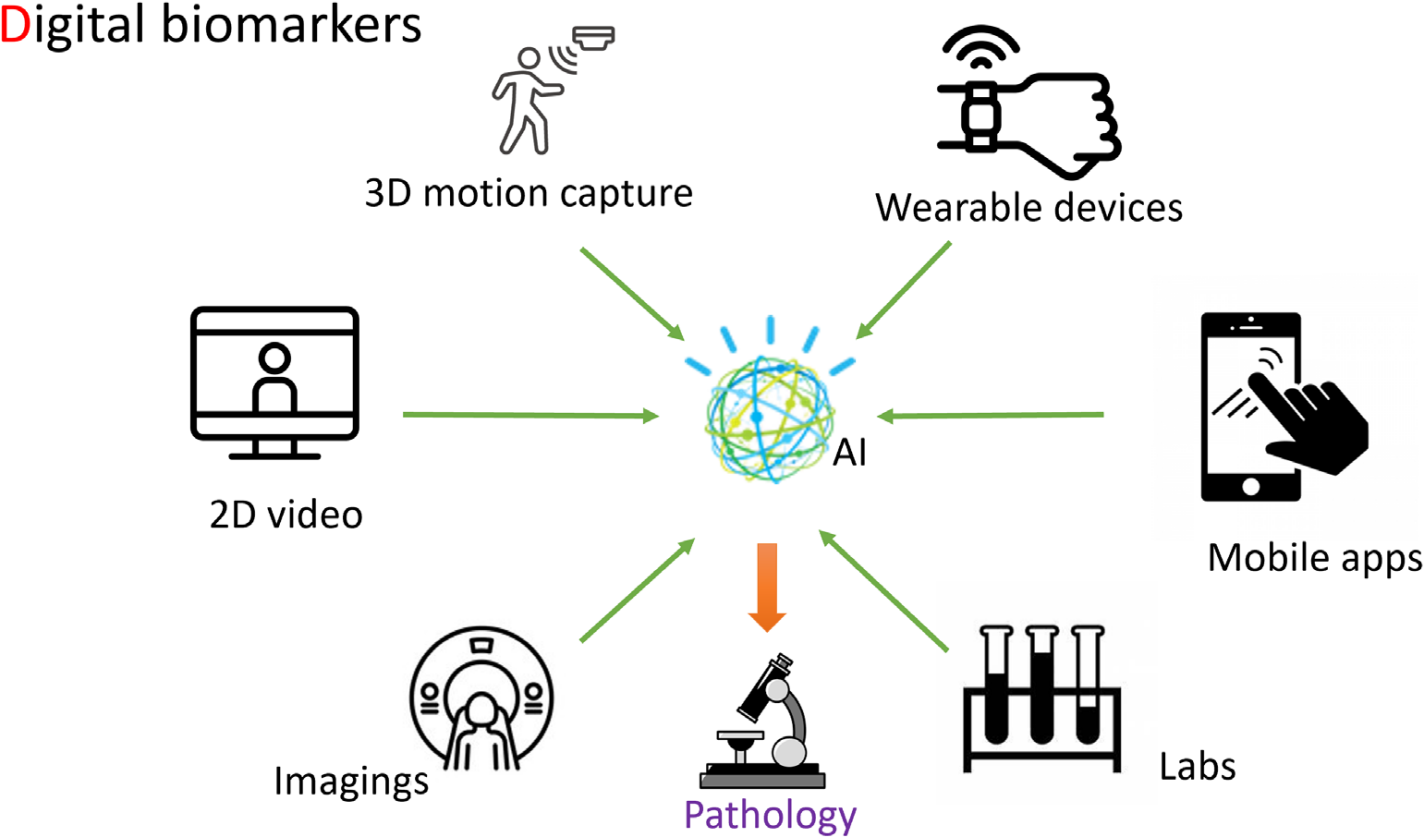
Digital biomarkers as a vital pillar. By integrating pathological diagnosis as the gold standard, AI has the potential to significantly improve diagnostic accuracy.

Advances in synuclein seed assays, such as real‐time quaking‐induced conversion (RT‐QuIC, have enabled the preclinical diagnosis of PD. These developments have sparked active discussions regarding the need to redefine PD from a biological perspective. Recent proposals to incorporate genetic information (G), neuroimaging (N), and synuclein assays (S) in addition to clinical status (C) into a new biological definition of PD underscore the importance of multi‐modal data integration.^21^ In this context, digital biomarkers (D) may emerge as a vital fifth pillar (Fig. 1). By integrating pathological diagnosis as the gold standard, rather than relying solely on clinical diagnosis, AI has the potential to significantly improve diagnostic accuracy. However, digital biomarkers face several challenges, including data noise, lack of standardization, the need for robust validation methods, and privacy concerns. Ensuring high data quality and harmonized methodologies is essential for their broader implementation.^22^, ^23^

In conclusion, digital tools are not replacing clinical judgment—they are augmenting it. By embedding AI and digital biomarkers within our phenomenological framework, we move toward a more precise model of Parkinsonism evaluation. This modern phenomenology may bridge the art and science of medicine, ensuring we remain both humanistic and data‐informed in our approach to care.

## Author Roles

(1) Research project: A. Conception, B. Organization, C. Execution; (2) Statistical Analysis: A. Design, B. Execution, C. Review and Critique; (3) Manuscript Preparation: A. Writing of the first draft, B. Review and Critique.

G.O.: 1A, 1B, 1C, 3A, 3B

## Disclosures

**Ethical Compliance Statement:** The author confirms that the approval of an institutional review board was not required for this work. Informed consent was not necessary for this work. The author confirms that they have read the Journal's position on issues involved in ethical publication and affirm that this work is consistent with those guidelines.

**Funding Sources and Conflict of Interest:** GO receives JSPS KAKENHI Grant Numbers 24K10667 and 21K12711. The author declares that there are no conflicts of interest relevant to this work.

**Financial Disclosures for the Previous 12 Months:** GO receives speaker honorarium from Medtronic Japan Co., Ltd., Boston Scientific Japan K.K., Abbott Japan Co., Ltd., Sumitomo Pharma Co., Ltd., Kyowa Kirin Co., Ltd., FP Pharmaceutical Corporation, Takeda Pharmaceutical Company Ltd., Eisai Co., Ltd., EA Pharma Co., Ltd., Ohtsuka Pharma, Ono Pharmaceutical Co., Ltd., Biogen Japan Ltd., Nihon Mediphysics Co., Ltd., Benesse Style Care Co., Ltd., and Abbvie.

## Data Availability

NA.
